# Clinical decisions and stigmatizing attitudes towards mental health problems in primary care physicians from Latin American countries

**DOI:** 10.1371/journal.pone.0206440

**Published:** 2018-11-15

**Authors:** Angel O. Rojas Vistorte, Wagner Ribeiro, Carolina Ziebold, Elson Asevedo, Sara Evans-Lacko, Jared W. Keeley, Daniel Almeida Gonçalves, Nataly Gutierrez Palacios, Jair de Jesus Mari

**Affiliations:** 1 Department of Psychiatry, Universidade Federal de São Paulo, São Paulo, Brazil; 2 London School of Economics and Political Science—Personal Social Services Research Unit, London, United Kingdom; 3 Global Mental Health Program, Columbia University, New York, United States of America; 4 King’s College London, Health Service and Population Research Department, Institute of Psychiatry, Psychology & Neuroscience, London, United Kingdom; 5 Department of Psychology, Virginia Commonwealth University, Virginia, United States of America; 6 Department of Preventive Medicine, Universidade Federal de São Paulo, São Paulo, Brazil; Centre Hospitalier Regional Universitaire de Tours, FRANCE

## Abstract

**Objective:**

The aim of this paper is to investigate how doctors working in primary health care in Latin American address patients with common mental disorders and to investigate how stigma can affect their clinical decisions.

**Methods:**

Using a cross-sectional design, we applied an online self-administered questionnaire to a sample of 550 Primary Care Physicians (PCPs) from Bolivia, Brazil, Cuba and Chile. The questionnaire collected information about sociodemographic variables, training and experience with mental health care. Clinicians’ stigmatizing attitudes towards mental health were measured using the Mental Illness Clinicians' Attitudes Scale (MICA v4). The clinical decisions of PCPs were assessed through three clinical vignettes representing typical cases of depression, anxiety and somatization.

**Results:**

A total of 387 professionals completed the questionnaires (70.3% response rate). The 63.7% of the PCPs felt qualified to diagnose and treat people with common mental disorders. More than 90% of the PCPs from Bolivia, Cuba and Chile agreed to treat the patients presented in the three vignettes. We did not find significant differences between the four countries in the scores of the MICA v4 stigma levels, with a mean = 36.3 and SD = 8.3 for all four countries. Gender (p = .672), age (p = .171), training (p = .673) and years of experience (p = .28) were unrelated to stigma. In the two multivariate regression models, PCPs with high levels of stigma were more likely to refer them to a psychiatrist the patients with depression (OR = 1.03, 95% CI, 0.99 to 1.07 *p*<0.05) and somatoform symptoms somatoform (OR = 1.03, 95% CI, 1.00 to 1.07, p<0.05) to a psychiatrist.

**Discussion:**

The majority of PCPs in the four countries were inclined to treat patients with depression, anxiety and somatoform symptoms. PCPs with more levels of stigma were more likely to refer the patients with depression and somatoform symptoms to a psychiatrist. Stigmatizing attitudes towards mental disorders by PCPs might be important barriers for people with mental health problems to receive the treatment they need in primary care.

## Introduction

Mental, neurological and substance use disorders (MNSD) exact a high toll, accounting for 13% of the total global burden of disease [1]. The burden of disease attributable to MNSD increased by 41% between 1990 and 2010. MNSD accounts for one in every 10 disability adjusted life years (DALYs) and affects disproportionally low- and middle-income countries (LMIC). Individuals with major depression and schizophrenia have a 40% to 60% greater chance of dying prematurely than the general population, owing to physical health problems that are often left untreated (e.g., cancer, cardiovascular diseases, diabetes and HIV infection) [2].

For instance, Ansseau et al. (2004) in a survey performed in Belgium found that, in the year prior to the study, 31% of adult patients in primary care services had affective disorders, 19% had some type of anxiety disorder, 18% had some type of somatoform disorder and 10% had disorders related to use of alcohol, and other studies estimate that 24% of patients who present to primary care physicians have a well-defined ICD-10 mental disorder [3].

This prevalence of mental disorders generates a demand for mental health care that is disproportionate to the number of specialists available in the health services [4]. Consequently, it has been proposed that most people with mental disorders, particularly those with common mental disorders, should be treated by non-specialist professionals in primary care (such as mental health providers) and specifically primary care physicians that not have a formal formation or specialization in mental health [4, 5].

In a systematic review of 63 studies on strategies to increase the availability of human resources for mental health care, Kakuma and colleagues note that primary care physicians (PCPs) who receive mental health training can efficiently identify, diagnose and treat mental disorders [6]. The authors also suggest that, if PCPs are engaged in the treatment of common mental disorders, the limited number of available mental specialists can be focused on the treatment of more complex clinical situations [6].

However, there are barriers that hinder the ability of primary care services to properly treat mental disorders. First, despite the high prevalence of psychiatric disorders in primary care populations, only 5.4% of patients seen by PCPs present with a psychiatric disorder as the main complaint [7, 8]. Second, a lack of training throughout one’s medical education, from undergraduate courses to specialization, makes it difficult for PCPs to identify mental disorders [9]. In addition to limiting the capacity for management in this population, the lack of training also contributes to the prevalence of misconceptions about mental disorders [9, 10], leading to stigma or prejudice among primary care providers towards mental disorders [11–16].

### Stigma as a barrier to mental health care

Stigma may be defined as a process involving labelling, separation, stereotype endorsement, prejudice and discrimination in a context in which social, economic or political power is exercised to the detriment of members of a social group [17]. It can be seen as an amalgamation of three related problems: a) lack of knowledge (ignorance and misinformation); b) negative attitudes (prejudice); and c) excluding or avoiding behaviours (discrimination) [15, 18–21]. The combination of these three elements is a powerful driver of social exclusion [15].

Health professionals who work in primary care and hold stigmatizing attitudes towards people with mental disorders are more pessimistic about patients´ adherence to the treatment of both mental and physical illnesses. Such professionals tend to make decisions based on mistaken assumptions or against standards of habitual care, based on their stigmatizing attitudes [16]. In this context, unreceptive attitudes of professionals who perpetuate stigmatizing beliefs tend to further distance people with mental disorders from the health care they need [22, 23].

In a systematic review carried out in Latin America the authors have found high levels of public and family stigma towards individuals affected by mental illness. These studies also found that stigma was associated with high levels of functional impairment among those who were stigmatized [24]. However, little is known about stigmatizing attitudes towards mental health illness among PCPs in Latin American countries and about the impact of stigma in the delivery of mental health care. Studies carried out in this area are scarce and have been conducted in the context of clinicians’ depression training programmes, assessing only the clinicians’ attitudes towards people with depression [19, 25].

Identifying stigmatizing attitudes of health professionals and the consequences of these attitudes for mental health care is a first step in order to propose interventions to reduce such attitudes. Thus, the main objective of this study was to evaluate how PCPs working in Latin American countries address patients with common mental disorders and to investigate how stigma related to mental disorders can affect the care offered to such patients.

## Method

### Setting

This study was carried out with PCPs from four Latin American countries: Chile, Cuba, Brazil and Bolivia.

1. **Chile**: With 17.91 million inhabitants and a gross national income (GNI) per capita of US$13,530, Chile is classified as a high-income country by the World Bank [26]. In 1993 and 2000, Chilean mental health plans established the incorporation of mental health in primary care as a priority to improve access to mental health care by facilitating an intersectional exchange of information and economic resources; replacing psychiatric hospitals with community-based services; and most importantly, strengthening the role of primary care centres in the provision of mental health treatment and care [27]. In 2013, Chile had 102 physicians and 7.23 psychiatrists per 100,000 inhabitants [28, 29].
2. **Cuba**: According to the World Bank, Cuba has a GNI per capita of US$ 6,570 and is classified as an upper-middle income country. The country has 11.48 million inhabitants, and like Chile, it has a long history of universal access to public health care [26]. The country also has a highly structured primary care network, which is the central pillar of the public health system. The implementation of Community Mental Health Centers and the reorientation of mental health care to primary care contributes to the development of more accessibility and community-based care for people with mental disorders [30]. Among the countries included in this study, Cuba has the largest number of physicians and psychiatrists per 100,000 inhabitants: 672 and 11, respectively [28].
3. **Brazil**: With 207.65 million inhabitants and GNI per capita of US$8,840, Brazil is also classified as an upper-middle income country [26]. Its legislation has guaranteed universal access to public health since the promulgation of the 1988 Constitution. Since then, several actions were proposed for the implementation of a service network able to meet the needs of the population. The Brazilian psychiatric reformation [30], resulted in a mental health treatment system in which psychiatric hospitals were replaced by a community-based mental health care network. This network included primary care services, which was considered the appropriate setting for treatment of common mental disorders. Brazil had 189 physicians and approximately four psychiatrists per 100,000 inhabitants [28, 29].
4. **Bolivia**: With 10,887,882 inhabitants and a GNI per capita of US$3,070, Bolivia is classified as a lower-middle income country [26]. In Bolivia, only since 2007 has the country had a National Universal Health Plan. As part of this National Universal Health Plan, the Bolivian government applied a National Mental Health Plan that is intended to guide the promotion, prevention, treatment and rehabilitation of mental illness. It is estimated, however, that 45% of the mental health services in primary care did not have physicians or mental health professionals on their teams [31, 32]. Bolivia had 47 physicians and 1.30 psychiatrists per 100,000 inhabitants, the lowest numbers of the four countries in the study [28, 29].

### Sample

We invited 550 PCPs from Bolivia (La Paz), Brazil (whole country), Cuba (*Mais Medicos programme* in Brazil) and Chile (Santiago) to participate in the study. Data collection was carried out between April and July 2016, through an online survey using the *Qualtrics* platform [33]. This is an online platform that permits the application of several questionnaires with a simple format for the participant. The PCP participants from Bolivia, Brazil and Chile lived and worked in their respective countries. In the case of Cuban professionals, they were part of the *Mais Medicos* programme in Brazil working in different regions. We obtained a list of Brazilian and Cuban PCPs from the health minister of Brazil, and in the case of Bolivian and Chilean PCPs from the members of our team through their contacts in primary care settings. Considering these aspects, our sample was considered as a convenience sample, principal based in the accessibility or proximity of the participants with the members of our research team. As a criterion for inclusion, in the first question of the questionnaire after agreeing to participate, was if the primary care physicians attended patients in primary care. Considering that in the four countries patients with common mental disorders are supposed to be treated in primary care settings, physicians who work in such settings are likely to encounter patients with common mental disorders in their clinical practice. It was not a requirement to treat mental health patients, but in the questionnaire it was consulted if they attended this type of patients.

### Instruments

We applied a questionnaire to obtain information related to gender, age and professional profile, including years of professional experience, years of formal training, level of knowledge in different areas of mental disorders, diagnostic systems in which they were trained and whether these classifications are useful for them to diagnose and to treat mental health problems in primary care.

The clinical decisions of mental health problems was evaluated using three clinical vignettes describing patients with symptoms of a) depression (V1); b) somatization (V2); and c) anxiety (V3) according to the ICD-10 Clinical Descriptions and Diagnostic Guidelines (CDDG) [34]. Clinical vignettes were developed by a group of psychiatrists who are members of the research team with extensive clinical experience, as well as two general practitioners working in the field of mental health. The vignettes describe the typical symptoms and/or complaints of patients with common mental disorders who attend primary care. Following each vignette, we asked the PCP to evaluate, using a four-item Likert scale, a) the presence of mental health symptoms that would require care; b) the decision to continue to treat the patient with a mental health problem in primary care versus referral to specialized care; and c) the decision to prescribe a psychotropic drug. The items in the Likert scale were: 1) strongly agree; 2) partially agree; 3) partially disagree; and 4) strongly disagree. Participants should choose one of the four possible alternatives for each of the possible clinical behaviours. Two of the three vignettes were randomly presented to each PCP.

Attitudes of primary care physicians in relation to patients with mental disorders were evaluated through the *Mental Illness*: *Clinicians' Attitudes Scale (MICA v4)*. MICA v4 is a scale originally developed to assess the attitudes of medical students towards people with mental disorders [35]. The scale was developed in England from focus groups with patients who utilize mental health services, caregivers of patients with mental disorders, medical students and psychiatry residents. The first version of the scale, drawn from the contents of the focus groups, contained 32 items. After a review of the content and as a result of the validation process, the 32 items were reduced to a total of 16 items, whose scores provide a general score of stigma. The 16 items are rated by means of a six-point Likert scale (from 1 = strongly agree to 6 = strongly disagree), with higher scores suggesting greater stigmatizing attitudes towards mental disorders. The questionnaire was translated and adapted into Portuguese and Spanish by the project team, following the steps proposed by Sartorius [36] (**S1 Appendix, S2 Appendix and S3 Appendix**).

The subjective perception of the capacity to diagnose and treat mental disorders was evaluated based on the following statement at the end of the questionnaire: "I feel capable enough to diagnose and treat patients with common mental disorders", followed by a Likert scale: 1) strongly agree; 2) partially agree; 3) partially disagree; and 4) strongly disagree.

### Data analysis

Data were analysed using the Statistical Package for Social Sciences (SPSS) for Windows, version 22. First, we calculated frequency distributions for categorical variables, as well as means and standard deviations for continuous variables. The primary outcomes were as follows: a) clinical behaviours of primary care professionals in relation to the clinical vignettes; and b) stigma levels according to the MICA v4 scale. The responses to the clinical vignettes were dichotomised into agreement (strongly agree and partially agree) or disagreement (strongly disagree and partially disagree) in order to facilitate the statistical analysis in comparison with the other variables of the study. We assessed the association of the clinical decisions variables with both stigma and sample characteristics through bivariate associations. We used 2X2 contingency tables when variables were categorical, Student’s *t*-tests when variables were continuous and ANOVA when variables were continuous in the case of the four countries categories. We assessed the impact of stigma on clinical decisions through logistical regression. **Model 1** estimated the associations between responses in the clinical vignettes and scores on the MICA v4; **Model 2** included the same variables from **Model 1** plus other potential confounding variables: gender, age, training and years of experience in primary care, as well as whether they felt able to treat and diagnose patients with mental disorders. For all statistical tests, we adopted a 5% significance level (*p*<0.05) and 95% of confidential interval.

### Ethics statements

This study design, and informed consent forms were reviewed and approved by the Comitê de Ética em Pesquisa da Universidade Federal de Sao Paulo (UNIFESP) (CEP Project / UNIFESP n1496 / 2015 Number: 51492815.5.0000.5505). Participants received detailed information about the research project and were enrolled with the freedom to choose to participate in the study or not. To warrant anonymity participants were de-identified in the database.

## Results

### Sample characteristics

387 professionals completed the questionnaires (70.3% response rate); 232 (59.9%) were female, and 155 (40.1%) were male. The mean age of the sample was 41.2 (SD = 9.71), ranging between 25 and 66 years, with a mean of 11.5 years of formal training (SD = 8.64), and 12 years of clinical experience (SD = 8.58) in primary care. No statistically significance differences between the countries were found between countries (F(3) = 1.46; p < .225). Cuban physicians had more years of training (F(3) = 15.7; p < .01, mean difference = 6.37) and more years of clinical experience (F(3) = 3.6; p < .01, mean difference = 2.83) than PCPs from Brazil (**Table 1**).

**Table 1:** Summary socio demographics variables and sample characteristics.

Variables	Brazil (N = 146)	Bolivia (N = 38)	Chile (N = 32)	Cuba (N = 171)	Total (N = 387)	p-value
**Gender**						
**Female**	95(65.1%)	19(50%)	21(65.6%)	97(56.7%)	232(59.9%)	.23
**Male**	51(34.9%)	19(50%)	11(34.4%)	74(43.3%)	155(40.1%)	
	**Mean (SD)**	**Mean (SD)**	**Mean (SD)**	**Mean (SD)**	**Mean (SD)**	
**Age**	40(11)	44(7)	40(10)	41(8)	41.2(9.7)	.17
**Training years**	8(9)	11(7)	11(8)	15(8)	11.5(8.5)	.01
**Experience Years**	11(10)	10(7)	12(8)	14(7)	12(8.6)	.01
**MICA Score**	36.1(8.6)	36.8(8.8)	37.7(8)	36(8.1)	37.8(8.3)	.18

The mental health areas with which professionals reported the greatest familiarity were as follows: Anxiety Disorders (87.3%), Depressive Disorders (83.4%), Disorders Related to Stress (58.6%) and Sleep Disorders (43.3%). Professionals were trained mainly on ICD-10 (63%) and DSM-IV (16%) diagnostic criteria, and 83.3% of the PCPs considered that using a classification system facilitates the diagnosis of mental health problems. Most professionals (63.7%) felt qualified to diagnose people with mental disorders.

### Clinical vignettes

Most of the PCPs (70%) recognized the presence of a mental illness in the three vignettes. In the depression, somatoform and anxiety vignettes, the majority of the professionals from Cuba, Chile and Bolivia (more than 90%) agreed to treat these patients. The PCPs from Brazil, however, were less prone to treat patients with depression in comparison with the PCPs from Cuba (X^2^(3) = 12.9, *p*<0.03). There were no between-country differences in the tendency to refer patients with anxiety and depression to psychiatrists. However, Brazilian physicians were less likely to refer the somatoform case to a psychiatrist (X^2^(3) = 8.9, *p*<0.02) (**Table 2**).

**Table 2:** Clinician’s recognition of mental disorders and decision making the clinical vignettes by countries.

		Bolivia N(% Total)		Brazil N(% Total)		Chile N(% Total)		Cuba N(% Total)		p-value (x^2)^
		Yes	No	Yes	No	Yes	No	Yes	No	
**Depression**	*Recognized a mental disorder*	16(6.6%)	7(2.9%)	62(25.4%)	31(12.7%)	14(5.7%)	9(3.7%)	68(27.9%)	37(15.2%)	.93
	*Refer to a Psychiatry*	16(6.8%)	8(3.4%)	47(20.1%)	33(14.1%)	15(6.4%)	9(3.8%)	65(27.8%)	41(17.5%)	.91
	*Willingness to treat in PHC*	23(9%)	-	87(34.1%)	11(4.3%)	22(8.6%)	1(0.4%)	110(43.1%)	1(0.4%)	.01
	*Antidepressant prescription*	21(8.5%)	2(0.8%)	72(29.1%)	18(7.3%)	20(8.1%)	3(1.2%)	97(39.3%)	14(5.7%)	.17
**Somatoform**	*Recognized a mental disorder*	23(9%)	2(0.8%)	68(26.7%)	30(11.8%)	13(5.1%)	9(3.5%)	77(30.2%)	33(12.9%)	.07
	*Refer to a Psychiatry*	20(7.8%)	5(2%)	53(20.8%)	45(17.6%)	16(6.3%)	6(2.4%)	76(29.8%)	34(13.3%)	.03
	*Willingness to treat in PHC*	21(8.2%)	4(1.6%)	86(33.7%)	12(4.7%)	18(7.1%)	4(1.6%)	97(38%)	13(5.1%)	.84
	*Antidepressant prescription*	22(8.6%)	3(1.2%)	87(34.1%)	11(4.3%)	20(7.8%)	2(0.8%)	95(37.3%)	15(5.9%)	.92
**Anxiety**	*Refer to a Psychiatry*	10(3.9%)	17(6.6%)	31(12%)	61(23.6%)	8(3.1%)	10(3.9%)	57(22.1%)	64(24.8%)	.25
	*Willingness to treat in PHC*	24(9.3%)	3(1.2%)	90(34.9%)	2(0.8%)	18(7%)	-	115(44.6%)	6(2.3%)	.17
	*Antidepressant prescription*	8(3.1%)	19(7.4%)	44(17.1%)	48(18.6%)	10(3.9%)	8(3.1%)	43(16.7%)	78(30.2%)	.10

### Implications for clinical decisions and levels of stigma

**Table 3** shows the associations between the responses to the vignettes and the demographic variables, clinical variables and stigma scores. In the depression vignette, the age of the PCP is related to antidepressant prescription (M = 41.7, SD = 9.26, *p* < .0.01, 95% CI, 0.43 to 7.44, mean difference = 3.93), older PCPs are more likely to prescribe an antidepressant. Similarly, training (M = 11.9, SD = 8.80, *p*<0.01, 95% CI, 3.64 to 13.3, mean difference = 8.46) and years of professional experience (M = 12.2, SD = 8.26, *p*<0.01, 95% CI, 01.97 to 11.1, mean difference = 6.55) were related to treating and prescribing an antidepressant (training years: M = 12.3, SD = 8.78, *p*<0.01, 95% CI, 0.59 to 6.69, mean difference = 6.55; and experience years: M = 12.4, SD = 8.25, *p*<0.01, 95% CI, 0.84 to 6.60, mean difference = 3.72). Professionals who felt capable dealing with and diagnosing mental disorders (63.7%) were more prone to treat depressive patients (X^2^(1) = 11.5, *p*<0.01). In the somatoform vignette, PCP age is related to deciding to treat these patients (M = 40.6, SD = 9.52, *p*<0.01, 95% CI, 0.13 to 7.63, mean difference = 3.87) and PCPs with more years of clinical experience were more likely to recognize a mental disorder (M = 15, SD = 9.59, *p*<0.01, 95% CI, 1.72 to 6.50, mean difference = 4.11). In the anxiety vignette, low levels of stigma are related with the decision to treat these patients (M = 35.6, SD = 8.02, *p*<0.01, 95% CI, 3.12 to 12.9, mean difference = 8.04). Those PCPs who had higher levels of stigma were more inclined to refer patients with depression (M = 37.4, SD = 8.03, *p*<0.01, 95% CI, 0.18 to 4.45, mean difference = 2.31) and somatoform symptoms (M = 37.7, SD = 8.82, *p*<0.01, 95% CI, 0.32 to 4.74, mean difference = 2.52) to a psychiatrist.

**Table 3:** Associations between the responses in the vignettes and demographic variables.

Variables	Vignette 1 (Depression) (%) p-value				Vignette 2 (Somatoform) (%) p-value				Vignette 1 (Anxiety) (%) p-value		
	Treat	Referral	Mental Disorder	Use of antidepressant	Treat	Referral	Mental Disorder	Use of antidepressant	Treat	Referral	Use of antidepressant
**Gender**											
** Female**	60.4%(0.33)	36.3%(0.89)	40.6%(0.89)	52.6%(0.85)	51.4%(0.71)	38%(0.79)	42.7%(0.78)	51.4%(0.44)	55.4%(0.77)	38%(0.02)*	24%(0.90)
** Male**	34.5%(0.33)	24.8%(0.89)	25%(0.89)	32.4%(0.85)	35.7%(0.71)	26.7%(0.79)	28.2%(0.78)	36.5%(0.44)	40.3%(0.77)	20.9%(0.02)	16.7%(0.90)
**I feel capable to treat mental disorders**	63.7%(0.01)*	58.5%(0.58)	61%(0.93)	61.8%(0.14)	63.7%(0.01)*	30.6%(0.03)*	63.7%(0.71)	63.7%(0.43)	64.5%(0.71)	64.5%(0.93)	64.5%(0.12)
	**t-Test (Mean) p-value**				**t-Test (Mean) p-value**				**t-Test (Mean) p-value**		
**Age**	41.1(0.09)	41.5(9.78)	40.3(9.44)	41.7(9.26)**	40.6(9.52)**	41.6(10.1)	40.3(9.8)	41.4(10.05)	41.2(8.78)	42(8.94)	41.2(9.75)
**Training Years**	11.9(8.80)**	11.9(8.68)	11.4(9)	12.3(8.78)**	11.1(8.51)	11.6(8.57)	10.8(8.41)	11.3(8.66)	12.2(8.79)	13(8.58)	12.2(9.33)
**Experience Years**	12.2(8.26)**	12.3(8.64)	11.5(8.34)	12.4(8.25)**	11.8(8.82)	12.4(9.32)	15(9.59)**	12.3(9.10)	12.4(8.46)	13.2(8.81)	12.2(9.48)
**Stigma score**	35.5(7.80)***	37.4(8.03)****	35.7(7.99)	36(7.93)	36.4(8.37)***	37.7(8.82)****	37.4(8.95)	36.7(8.63)	35.6(8.02)***	36.4(8.45)	36.3(8.67)

Notes

The minimum stigma score was 16 and the maximum 80.

* x^2^ p-value<0.05.

** t-Test p-value<0.05 (More age, training years and experience years)

*** t-Test p-value<0.05 (Low levels of Stigma)

****t-Test p-value<0.05 (High levels of Stigma)

We did not find significant differences between the four countries in the scores of the MICA v4 stigma levels having a mean = 36,3 and SD = 8,3 for all four countries. Gender (p < .672), age (r = .072, *p* = 0.171), training (r = .022, *p* = 0.673) and years of experience (r = .055, *p* = 0.28) were unrelated to levels of stigma. When we compared the MICA v4 score with the groups of professionals who felt able to treat mental health disorders in primary care and those who felt unprepared, the results showed a significant difference in the scores for that PCPs who felt prepared (M = 35.7, SD = 8.43) and felt unprepared (M = 38.5, SD = 7.56), (t(385) = 2.5, *p*<0.01, 95% CI 0.61 to 4.86). PCPs professionals who considered themselves unprepared have higher levels of stigma than physicians who consider themselves prepared.

**Table 4** shows the logistic regressions of the association between the responses of the vignettes and the stigma levels (**Model 1**), as well as the multivariate regression (**Model 2**), which included the same variables from the **Model 1** plus gender, age, training and years of experience, as well as whether the PCP felt qualified to diagnose and treat common mental disorders. In **Model 1** when the depression (OR = 0.93, 95% CI, 0.87 to 0.99, *p*<0.03) and somatoform vignette (OR = 0.95, 95% CI, 0.92 to 0.99, *p*<0.03) were considered, higher stigma levels reduced the odds of treat these patients. Also, the PCPs with higher levels of stigma were more likely to refer to a psychiatrist the patients with depression (OR = 1.03, 95% CI, 1.00 to 1.07, *p*<0.01) and somatoform (OR = 1.03, 95% CI, 1.00 to 1.07, *p*<0.03). In the anxiety vignette, higher stigma levels reduced the odds of treat these patients (OR = 0.90, 95% CI, 0.85 to 0.97, *p*<0.03). When we included the other variables (**Model 2**), only the associations for depression (OR = 1.03, 95% CI, 0.99 to 1.07 *p*<0.05) and somatoform (OR = 1.03, 95% CI, 1.00 to 1.07, p<0.05) vignettes remained significant where professionals with higher levels of stigma were more likely to refer them to a psychiatrist. These means that PCPs with more levels of stigma were more likely to refer the patients with depression and somatoform symptoms to a psychiatrist.

**Table 4:** Associations between vignettes responses and stigma levels (MICA v4 scores) through logistic regression models.

	Model 1*		Model 2**		
	O.R. (95% CI)	p	O.R. (95% CI)		p
*Depression*					
*Willingness to treat in PHC*					
**Stigma Score**	**0.93(0.87–0.99)**	**0.03**	**Stigma Score**	**0.91(0.85–0.99)**	**0.02**
			Nationality	1.61(0.61–4.19)	0.33
			Gender	0.68(0.17–2.82)	0.60
			Age	0.97(0.90–1.03)	0.36
			**Training years**	**1.40(1.05–1.86)**	**0.02**
			Experience years	1.00(0.89–1.13)	0.98
*Refer to a Psychiatry*					
**Stigma Score**	**1.03(1.00–1.07)**	**0.04**	**Stigma Score**	**1.03(0.99–1.07)**	**0.05**
			Nationality	**0.99(0.76–1.30)**	**0.97**
			Gender	1.09(0.61–1.95)	0.76
			Age	0.99(0.95–1.03)	0.62
			Training years	0.99(0.95–1.05)	0.92
			Experience years	1.03(0.98–1.09)	0.24
*Recognized a mental disorder*					
**Stigma Score**	**0.99(0.96–1.03)**	**0.64**	**Stigma Score**	**0.99(0.95–1.02)**	**0.41**
			Nationality	0.93(0.71–1.22)	0.59
			Gender	0.88(0.49–1.57)	0.68
			Age	0.99(0.95–1.03)	0.64
			Training years	1.02(0.97–1.06)	0.51
			Experience years	0.99(0.94–1.05)	0.85
*Antidepressant prescription*					
**Stigma Score**	**1.02(0.97–1.07)**	**0.39**	**Stigma Score**	**1.01(0.96–1.06)**	**0.75**
			Nationality	1.00(0.68–1.49)	0.99
			Gender	1.42(0.61–3.34)	0.42
			Age	1.01(0.95–1.07)	0.79
			Training years	1.06(0.98–1.14)	0.13
			Experience years	1.02(0.94–1.10)	0.66
**Somatoform**					
*Willingness to treat in PHC*					
**Stigma Score**	**0.95(0.92–0.99)**	**0.03**	**Stigma Score**	**0.95(0.91–0.99)**	**0.03**
			Nationality	1.03(0.70–1.51)	0.88
			Gender	1.33(0.58–3.05)	0.49
			Age	0.96(0.91–1.02)	0.18
			Training years	1.00(0.95–1.06)	0.93
			Experience years	0.99(0.93–1.06)	0.91
*Refer to a Psychiatry*					
**Stigma Score**	**1.03(1.00–1.07)**	**0.03**	**Stigma Score**	**1.03(1.00–1.07)**	**0.05**
			Nationality	1.17(0.90–1.52)	0.25
			Gender	1.08(0.62–1.89)	0.79
			Age	1.01(0.97–1.06)	0.59
			Training years	0.99(0.95–1.04)	0.81
			Experience years	1.01(0.96–1.06)	0.81
*Recognized a mental disorder*					
**Stigma Score**	**1.02(0.99–1.06)**	**0.14**	**Stigma Score**	**1.02(0.98–1.05)**	**0.36**
			Nationality	0.89(0.68–1.18)	0.43
			Gender	1.01(0.56–1.83)	0.98
			Age	1.01(0.97–1.06)	0.60
			Training years	1.01(0.97–1.06)	0.56
			**Experience years**	**0.94(0.89–0.99)**	**0.01**
*Antidepressant prescription*					
**Stigma Score**	**0.99(0.95–1.03)**	**0.68**	**Stigma Score**	**0.99(0.94–1.03)**	**0.59**
			Nationality	0.91(0.62–1.34)	0.64
			Gender	1.20(0.53–2.74)	0.66
			Age	1.05(0.98–1.14)	0.15
			Training years	0.96(0.88–1.03)	0.26
			Experience years	0.98(0.93–1.09)	0.86
**Anxiety**					
*Willingness to treat in PHC*					
**Stigma Score**	**0.90(0.85–0.97)**	**0.01**	**Stigma Score**	**0.89(0.83–0.97)**	**0.01**
			Nationality	0.92(0.50–1.69)	0.79
			Gender	2.34(0.53–10.36)	0.26
			Age	0.93(0.85–1.01)	0.09
			Training years	1.04(0.95–1.15)	0.41
			Experience years	1.02(0.92–1.14)	0.68
*Refer to a Psychiatry*					
**Stigma Score**	**1.01(0.98–1.04)**	**0.42**	**Stigma Score**	**1.01(0.98–1.04)**	**0.57**
			Nationality	1.11(0.86–1.42)	0.44
			**Gender**	**1.85(1.08–3.18)**	**0.03**
			Age	0.99(0.95–1.04)	0.72
			Training years	1.01(0.97–1.05)	0.60
			Experience years	1.02(0.97–1.07)	0.51
*Antidepressant prescription*					
**Stigma Score**	**1.01(0.98–1.04)**	**0.48**	**Stigma Score**	**1.01(0.98–1.05)**	**0.51**
			Nationality	0.84(0.65–1.08)	0.17
			Gender	0.79(0.46–1.36)	0.39
			Age	0.99(0.96–1.05)	0.98
			Training years	0.99(0.96–1.04)	0.88
			Experience years	0.99(0.95–1.05)	0.97

* Model 1 = bivariate between Vignette responses and MICA scores.

** Model 2 = Model 1 plus potential confounders (gender, nationality, age, training and experience years).

Abbreviations: CI = confidence interval; p = p value.

Stigma score: continuous variable. (higher scores mean higher stigma).

Reference category: Agreed.

## Discussion

The majority of PCPs in the four countries were inclined to treat patients with depression, anxiety and somatoform symptoms. Despite their disposition to treat these disorders, a considerably high proportion of PCPs also reported that they would refer these patients to a psychiatrist. There is evidence that physicians stigmatizing attitudes towards mental disorders might be an important barrier for people with mental health problems to receive the treatment they need [37, 38]. Gender, age, training and years of experience in primary care were unrelated to having higher or lower levels of stigma in our sample when compared with other studies that show other results [13, 39, 40]. It is suggested that doctors’ self-efficacy for recognizing and managing depression was likely to influence the care depressed patients received from their PCP [41]. Stigmatizing attitudes were associated with not feeling prepared to care for patients with common mental disorders in our sample.

Lower levels of knowledge, lack of adequate training and stigmatizing attitudes were associated with poorer diagnostic and treatment efficacy in mental disorders [42–49]. After adjusting for potential confounders, our regression model showed that stigma was related to a higher likelihood of professionals referring patients with depression and somatic symptoms. In general, the PCPs recognized the mental disorders presented in the vignettes and felt able, in principle, to treat these patients, but despite their affirmation that they have more knowledge regarding depression or anxiety relative to somatoform cases, the majority of PCPs would refer these patients to a psychiatrist.

Many studies showed that PCPs have more negative attitudes towards mental illness when compared to other professionals and to the general population. Studies comparing such groups found that PCPs had the highest levels of stigmatizing attitudes, followed by other primary care professionals, mental health professionals and the general population [13, 37, 38, 40]. Thus, it is important to identify ways to increase the effectiveness of PCPs in managing depression and other mental disorders, as most depressed patients (and others mental disorders) seek treatment in primary care and are frequently undertreated. Therefore, PCPs represent a potential target for interventions to reduce the disparities in the care of individuals with mental disorders [39, 43, 50, 51]. Interventions need to consider the context of the primary care setting and consider barriers to treatment including lack of adequate time, training, competing agendas, and lack of adequate reimbursement. Ineffective treatment leads to patient and physician frustration due to a lack of progress [50].

This study has several limitations: a) the sample is only a representation of the professionals of the four countries who had access to the internet, and may not be representative of their countries; b) the PCPs from Cuba were trained in their country, but at the moment of the study, they worked in Brazil; and c) the vignettes presented are based on written cases presenting symptoms of three common mental disorders, which may not represent live interactions with a patient.

The main conclusions of this study were that lower levels of knowledge, a lack of adequate training and stigmatizing attitudes towards mental disorders might be important barriers for people with mental health problems to receive the treatment they need. Gender, age, experience and years of training in the area of primary care are unrelated to the professionals’ levels of stigma. Stigmatizing attitudes are associated with not feeling prepared to care for patients with common mental disorders in our sample. Additionally, levels of stigma are associated with referring patients to a psychiatrist for depression and somatoform cases. Thus, PCPs need more training and adequate tools to address mental disorders, such as a user-friendly, pragmatic classification system that addresses the high prevalence of mental disorders in primary care and community settings [52].

## Supporting information

S1 Appendix Mental Illness: Clinicians' Attitudes Scale (MICA v4) in English. (PDF)

S2 Appendix Mental Illness: Clinicians' Attitudes Scale (MICA v4) in Spanish. (PDF)

S3 Appendix Mental Illness: Clinicians' Attitudes Scale (MICA v4) in Portuguese. (PDF)
