# Supplementary material for: Clinical decisions and stigmatizing attitudes towards mental health problems in primary care physicians from Latin American countries
Source: PLoS One. 2018 Nov 15;13(11):e0206440. doi: 10.1371/journal.pone.0206440 (PMC6237310; doi:10.1371/journal.pone.0206440)
Supplement: S2 Appendix — (PDF) [file pone.0206440.s002.pdf]

Primary Care Physicians Spanish

Q1 Estimado (a) colega lo (a) invitamos a que participe en el presente estudio sobre temas referentes al tratamiento de los trastornos mentales en la atención primaria (básica), toda la información proporcionada contará con la máxima confidencialidad, contribuyendo al desarrollo de tres líneas de investigación que se vienen desarrollando en la Universidad Federal de São Paulo (UNIFESP), Departamento de Psiquiatría, en 4 países: Brasil, Bolivia, Chile, Cuba.

Q2 Soy Médico de Atención Primaria calificado para ejercer en mi país, y estoy de acuerdo en brindar información referente a las preguntas realizadas en el presente cuestionario los cuales contribuirán a mejorar la atención de los pacientes con trastornos mentales en la atención primaria (Básica) de Salud.

- ❍ Si (1)
- ❍ No (2)

Q3 Actualmente, ¿atiende a pacientes como parte de sus actividades profesionales?

- ❍ Si (1)
- ❍ No (2)

Q4 Género

- ❍ Femenino (1)
- ❍ Masculino (2)

Q5 ¿En qué ciudad trabaja?

Q6 Nacionalidad

Q7 Año de Nacimiento

Q8 ¿Qué lenguas usted usa más frecuentemente en su vida profesional?

- ❑ Inglés (1)
- ❑ Portugués (2)
- ❑ Español (3)

Q9 ¿Cuál es su especialidad?

Q10 ¿Cuántos años de entrenamiento profesional formal usted tiene, sin incluir los años de estudios universitarios? (Note que nos referimos apenas a los años de formación, no de experiencia. Formación formal incluye posgraduación, estadios de especialización o post doctorado, mas no incluye educación profesional continuada)

Q11 Como parte de su formación profesional (académico y/o clínico), ¿en cuáles de los siguientes sistemas diagnósticos para trastornos mentales o comportamentales usted fue entrenado para utilizar?:

- ❑ CIE-10 (Clasificación Internacional de Enfermedades 10ma Revisión) (1)
- ❑ CIE-9 (Clasificación Internacional de Enfermedades 9na Revisión) (2)
- ❑ CIE-8 (Clasificación Internacional de Enfermedades 8va Revisión) (3)
- ❑ DSM-IV (Manual Diagnóstico y Estadístico de Enfermedades Mentales 4ta Edición) (4)
- ❑ DSM-IV-TR (Manual Diagnóstico y Estadístico de Enfermedades Mentales 4ta Edición-Revisada) (5)
- ❑ DMS-III (Manual Diagnóstico y Estadístico de Enfermedades Mentales 3ra Edición) (6)
- ❑ Ninguno (7)

Q12 Actualmente, ¿cuáles son sus contextos de trabajo principales? Entendemos por contexto de trabajo principal el local donde trabaja 5 horas o más por semana (Marque todos que se aplican)

- ❑ Cuidados de Salud Primarios (1)
- ❑ Otros contextos médicos generales (incluyendo centros médicos universitarios, facultad/escuela de medicina) (2)
- ❑ Servicios de salud mental (incluyendo servicios en las universidades, departamentos hospitalarios y clínicas ambulatorias) (3)
- ❑ Programas especializados de tratamiento de abuso de substancias. (4)
- ❑ Consultorio particular (sólo o en grupo) (5)
- ❑ Universidad (no en servicio de tratamiento médico, de salud mental o abuso de substancias) (6)
- ❑ Administración de salud pública o agencia del gobierno. (7)
- ❑ Organización no gubernamental (ONG) (8)
- ❑ Otros (9)

Q13 ¿Cuántos años de experiencia formal tiene en su especialidad?

Q14 Por favor, indique cuál de los siguientes tipos de servicios de salud mental ofrece personalmente a los pacientes como parte de sus actividades profesionales regulares (marque todos que se correspondan):

- ❑ Evaluación diagnóstica de los trastornos mentales y comportamentales. (1)
- ❑ Evaluación diagnóstica y supervisión de sustancias psicoactivas. (2)
- ❑ Evaluación psicológica (Por ejemplo: evaluación neuropsicológica, tests de personalidad, etc) (3)
- ❑ Psicoterapia (4)
- ❑ Psicoeducación (5)
- ❑ Otros (especificar): (6) ____________________
- ❑ Ninguno (7)

Q15 ¿Supervisa directamente los servicios de atención de salud prestados por otras personas? (Por supervisión directa queremos decir que usted supervisa o controla los servicios prestados a través de mecanismos tales como supervisión cara a cara,

conferencias o discusión de casos, revisión de historias clínicas, y que es directamente responsable por la calidad de los servicios clínicos ofrecidos)

- ❍ Si (1)
- ❍ No (2)

Q16 ¿Cuántas personas supervisa directamente en la prestación de servicios de salud?

Q17 Por favor indique ¿cuáles de los siguientes tipos de servicios de salud mental son ofrecidos por las personas que supervisa? (Marque todos que se correspondan)

- ❑ Evaluación diagnóstica de los trastornos mentales y comportamentales. (1)
- ❑ Evaluación diagnóstica y supervisión de sustancias psicoactivas. (2)
- ❑ Evaluación psicológica (Por ejemplo: evaluación neuropsicológica, tests de personalidad, etc) (3)
- ❑ Psicoterapia (4)
- ❑ Psicoeducación (5)
- ❑ Otros (especificar): (6) ____________________
- ❑ Ninguno (7)

Q18 En una semana típica, ¿en qué tipo de contextos ofrece o supervisa servicios de salud? (Marque todos los que correspondan):

- ❑ Servicio de Salud Mental (1)
- ❑ Hospital (2)
- ❑ Centro de atención primaria (3)
- ❑ Domiciliar (4)
- ❑ Hospitalización parcial/Hospital de Día (5)
- ❑ Clínica privada (6)
- ❑ Consultorio particular (7)

Q19 En una semana típica, ¿en qué tipo de contextos ofrece o supervisa servicios de salud? (Marque todos los que correspondan): Programa especializado para tratamiento de abuso de sustancias:

- ❑ Hospital (1)
- ❑ Centro de atención primaria (2)
- ❑ Domiciliar (3)
- ❑ Hospitalización parcial/Hospital de Día (4)
- ❑ Clínica privada (5)
- ❑ Consultorio particular (6)
- ❑ No se aplica (7)

Q20 En una semana típica, ¿en qué tipo de contextos ofrece o supervisa servicios de salud? (Marque todos los que correspondan): Contextos médicos generales:

- ❑ Hospital (1)
- ❑ Centro de atención primaria (2)
- ❑ Domiciliar (3)
- ❑ Hospitalización parcial/Hospital de Día (4)
- ❑ Clínica privada (5)
- ❑ Consultorio particular (6)
- ❑ No se aplica (7)

Q21 En una semana típica, ¿en qué tipo de contextos ofrece o supervisa servicios de salud? (Marque todos los que correspondan): Otros contextos:

- ❑ Prisiones u otros centros de detención (1)
- ❑ Programas comunitarios (2)
- ❑ Programas escolares (3)
- ❑ Centros Universitarios de salud mental o psicología/Consejería (4)
- ❑ Otros (5)
- ❑ No se aplica (6)

Q22 ¿En qué tipos de comunidades están localizados los contextos de servicio que ofrece o supervisa? (Si ofrece servicios en más de un ambiente o espacio, marque todos los que se correspondan)

- ❑ Grandes centros urbanos (1)
- ❑ Suburbio de un gran centro urbano (2)
- ❑ Ciudad de tamaño medio (3)
- ❑ Ciudad pequeña (4)
- ❑ Villa (5)
- ❑ Ambiente rural (6)

Q23 Para los servicios de salud que ofrece o supervisa, ¿cuál es el porciento de pacientes en cada grupo etario? (Los porcientos tienen que totalizar 100. Si alguno de ellos es 0, debe marcarlo también)

______ Niños y/o Niñas (0-12) (1)
______ Adolescentes (12-18) (2)
______ Adultos (18-65) (3)
______ Adultos mayores (más de 65) (4)

Q24 ¿Quién es, más frecuentemente, el responsable por la atribución del diagnóstico psiquiátrico a los pacientes que atiende o supervisa?

- ❍ Yo o alguien bajo mi supervisión directa atribuye, habitualmente, el diagnóstico. (1)
- ❍ Otro profesional de salud que no está bajo mi supervisión atribuye, habitualmente, el diagnóstico (Por ejemplo: otro médico del servicio) (2)
- ❍ Los diagnósticos son, habitualmente, atribuidos por los registros médicos o por los codificadores (Historias Clínicas del paciente) (3)
- ❍ No son, habitualmente, atribuidos diagnósticos psiquiátricos. (4)
- ❍ Otros (5)

Q25 Por favor, indique con qué regularidad utiliza los siguientes sistemas de clasificación en su práctica clínica o supervisión en relación a los Trastornos de Salud Mental:

	Rutinariamente (8)	Frecuentemente (9)	A veces (6)	Raramente (10)	Nunca (7)
CIE-9 o CIE-9-CM (1)	❍	❍	❍	❍	❍
CIE 10 (2)	❍	❍	❍	❍	❍
DSM IV o DSM-IV-TR (3)	❍	❍	❍	❍	❍
Otros (4)	❍	❍	❍	❍	❍

Q26 En función del sistema de clasificación que usted usa más frecuentemente en su práctica clínica, ¿considera que la clasificación facilita su tarea de diagnosticar pacientes con problemas de salud mental en la atención primaria?

❍ Si (1)
❍ No (2)

Q27 El sistema de clasificación que usted usa más frecuentemente en relación a los trastornos de salud mental, ¿se ajusta a la sintomatología que presentan sus pacientes en su práctica clínica en la atención primaria?

❍ Si (1)
❍ No (2)

Q28 ¿Usted tiene responsabilidad administrativa por una o más unidades que ofrecen servicios de salud mental, aunque no ofrezca personalmente cuidados clínicos directos o supervisión?

❍ Si (1)
❍ No (2)

Q29 ¿Cuántas personas ofrecen servicios de salud mental en las unidades de las cuales usted es responsable?

Q30 En una semana típica, ¿cuántas horas usted dedica a las siguientes actividades profesionales? (Si para alguna actividad es "0", no olvide marcarlo también)
______ Ofreciendo directamente servicios de salud mental a pacientes (ej: evaluaciones, terapias psicológicas o comportamentales, gestión o manejo de la medicación) (1)
______ Ofreciendo otros servicios de cuidados de salud (no de salud mental) (2)
______ Supervisión de servicios de salud ofrecidos por otros. (3)
______ Enseñanza o educación. (4)
______ Investigación (5)
______ Administración (6)
______ Otros (especifique) (7)

Q31 Por favor, seleccione hasta tres áreas relacionadas con los trastornos mentales y comportamentales de la CIE-10 que tenga más conocimiento y experiencia:
- ❑ Demencia, Delirio, y trastornos relacionados (1)
- ❑ Trastornos relacionados al uso de sustancias (2)
- ❑ Esquizofrenia y trastornos relacionados (3)
- ❑ Trastornos del humor (4)
- ❑ Trastornos de ansiedad (5)
- ❑ Trastornos relacionados al estrés (6)
- ❑ Trastorno obsesivo compulsivo y trastornos relacionados (7)
- ❑ Trastornos somatomorfos (8)
- ❑ Trastornos alimentarios (9)
- ❑ Trastornos del sueño (10)
- ❑ Trastornos sexuales (11)
- ❑ Trastornos de personalidad (12)
- ❑ Deficiencias intelectuales (13)
- ❑ Trastornos del espectro autista (14)
- ❑ Déficit de atención y Trastorno de conducta (15)
- ❑ Epidemiología (16)
- ❑ Salud Pública (17)
- ❑ Neurociencia (18)
- ❑ Otros (Especificar) (19) ____________________

Q32 Yo me siento suficientemente capacitado para diagnosticar y tratar los pacientes con transtornos mentales comunes:
- ❍ Concuerdo Plenamente (1)
- ❍ Concuerdo Parcialmente (2)
- ❍ Desacuerdo Parcialmente (3)
- ❍ Desacuerdo Plenamente (4)

Q33 Por favor, ofrezca cualquier comentario adicional sobre sus contextos laborales, intereses o especialidad que considere no fueron abordados adecuadamente en este cuestionario.

Q34 A continuación se presentan dos casos clínicos, por favor marque la opción que considere en cada inciso o caso:

Q35 Identificación de caso Clínico: MDS, sexo femenino, 53 años, llega a la Unidad Básica de Salud (Consultorio Médico de la Familia/Área de Atención Primaria) con queja de “mareos”. Historia Actual: Es diabética, previamente compensada. Niega náuseas, xerostomía, dolor abdominal o síntomas urinarios. Dice que el mareo comenzó ayer (en la víspera), pero ha ido empeorando progresivamente. La enfermera pregunta si ella “pasó por alguna alteración nerviosa” y la paciente comienza a llorar. Dice que frecuentemente tiene mareo, que empeora cuando discute con el hijo. Informaciones Adicionales: Desde que su hijo comenzó a “hacer cosas malas por causa de las malas compañías”, hace cerca de un año está sin deseos de salir de la cama. Llora varias veces al día y cree que es una persona triste. Está comiendo poco, pues luego se siente “llena” y con náuseas, y tiene muchas pesadillas. En el examen se nota que la paciente está hidratada, sus exámenes abdominal y neurológico son normales. La presión alterial es de 140x90 mmHg y la glicemia de 248 mg/dL.
a) Responda las siguientes preguntas con la conducta a seguir en relación al caso:

	Concuerdo Plenamente (1)	Concuerdo Parcialmente (2)	Desacuerdo Parcialmente (3)	Desacuerdo Plenamente (4)
La paciente debe ser tratada por una complicación aguda de la diabetes. (1)	❍	❍	❍	❍
Le debe ser administrada una benzodiazepina y se le puede dar el alta. (2)	❍	❍	❍	❍
MDS puede tener un trastorno mental importante. (3)	❍	❍	❍	❍
El Clínico/Médico de Familia en la comunidad debe tratar la diabetes y remitir a la paciente para un psiquiatra. (4)	❍	❍	❍	❍
MDS presenta un cuadro de depresión que debe, en principio, ser tratado por el	❍	❍	❍	❍

Clínico/Médico de Familia. (5)				
MDS presenta síntomas de ansiedad que no necesitan tratamiento. (6)	❍	❍	❍	❍
MDS tiene una depresión importante que el Clínico/Médico de Familia debe tratar con un antidepresivo. (7)	❍	❍	❍	❍

Q36 Identificación Caso: L.C.P., 54 anos, sexo femenino, llega para consulta marcada con queja de “dolor en los brazos”. Historia Actual: Dice que tiene el síntoma desde los 6 años, con períodos de empeoramiento, además de “crisis de hormigueo” en el hemisferio derecho, que duran de 2 a 4 horas, por lo menos 2 veces por semana. También refiere “cansancio exagerado” y períodos de plenitud gástrica (empacho). Trae para la consulta exámenes realizados en diferentes servicios en el último año: hemograma, glicemia, lipemia, electrolitos séricos, enzimas hepáticas y hormonas tiroideas, endoscopia, tomografías de cráneo y columna, doppler de arterias carótidas y vertebrales y electroneuromiografía, todos sin alteraciones. Informaciones Adicionales: Hace 4 años que está apartada del trabajo, desde el fallecimiento de su hijo. Dice que no le gusta salir de casa, no tiene deseos de encontrarse con sus familiares o amigos y solo tiene relaciones sexuales con el marido “por obligación”. Percibe que sus síntomas empeoran mucho cuando está nerviosa. Usa anti-inflamatorios no esteroides, analgésicos y relajantes musculares casi diariamente, sin una mejora significativa. Niega la presencia de dolencias crónicas. En la consulta presenta presión arterial de 120x75mmHg, dolor a la palpación muscular en el trapecio y romboides, sin limitación de movimientos. Examen neurológico: fuerza, tono muscular, reflejos, propio percepción y coordinación normal en miembros superiores e inferiores. a) Responda las siguientes preguntas con la conducta a seguir en relación al caso:

	Concuerdo Plenamente (1)	Concuerdo Parcialmente (2)	Desacuerdo Parcialmente (3)	Desacuerdo Plenamente (4)
La paciente debe recibir un estudio clínico más profundo para identificar una enfermedad física. (1)	❍	❍	❍	❍
Debe ser administrado para la paciente un benzodiazepínico para uso continuo. (2)	❍	❍	❍	❍
LCP puede tener un trastorno mental importante. (3)	❍	❍	❍	❍
LCP se beneficiaría con el uso de anti-depresivos. (4)	❍	❍	❍	❍
El Clínico/Médico de Familia y Comunitario debe remitir a la paciente para un	❍	❍	❍	❍

psiquiatra. (5)				
LCP presenta un trastorno mental que debe, en principio, ser tratado por el Clínico/Médico de Familia. (6)	❍	❍	❍	❍
LCP no presenta una dolencia orgánica, por tanto, no necesita tratamiento. (7)	❍	❍	❍	❍

Q37 Identificación Caso: A.C.V., 46 años, sexo masculino, viene para la consulta con queja de dolor en el pecho. Historia Actual: Refiere dolor en el hemitórax izquierdo, no relacionado con esfuerzo, con inicio hace 4 meses con un aumento progresivo en la frecuencia de aparición desde entonces. Algunos de estos episodios son acompañados de disnea, y otros como parestesia en las manos. Refiere ocasionalmente la presencia de temblores y mareo. Cuando se le pregunta dice que tiene problemas para dormir. Se ha sentido irritado y discute mucho con su esposa, pero no cree que los síntomas tengan relación con su estado emocional. Informaciones Adicionales: Cuenta que tiene un hijo de 24 años que se va a casar en un mes y que su hija de 19 años quiere ir a la universidad, pero no sabe si va a poder pagarlo. Hipertenso, en uso de Hidroclorotiazida 25mg por la mañana y Enalapril 5mg cada 12 horas, niega antecedentes familiares de dolencias (enfermedades) cardiovasculares. Comerciante, trabaja cerca de 12 horas por día, no fuma. En el examen: presión arterial 110x70, peso 75kg, altura 1,68m (IMC 25,5), la auscultación cardíaca y pulmonar sin alteraciones (cambios). Trae resultados de exámenes complementarios realizados el mes anterior: glicemia 99 mg/dL, colesterol total 223, HDL 65, triglicerideos 145, creatinina 0,9, potasio 3,8, orina sin proteinuria e eletrocardiograma con ritmo sinusal, sin alteraciones (anormalidades). a) Responda las siguientes preguntas con la conducta a seguir en relación al caso:

	Concuerdo Plenamente (1)	Concuerdo Parcialmente (2)	Desacuerdo Parcialmente (3)	Desacuerdo Plenamente (4)
El paciente solo precisa un estudio para un síndrome coronario. (1)	❍	❍	❍	❍
Se aconseja al paciente el uso de antidepresivo. (2)	❍	❍	❍	❍
ACV tiene indicado el uso de un benzodiazepínico. (3)	❍	❍	❍	❍
El Clínico/Médico de Familia y comunitario debe remitir al paciente para un psiquiatra. (4)	❍	❍	❍	❍
ACV presenta un cuadro de ansiedad que debe, en principio, ser tratado por el Clínico/Médico de Familia. (5)	❍	❍	❍	❍

ACV no precisa de ningún otro tratamiento más que el actual para la hipertensión. (6)	❍	❍	❍	❍

Q38 Instrucciones: Por cada una de las preguntas del número 1 a la 16, por favor responda marcando una sola casilla. La enfermedad mental se refiere aquí a las condiciones por las cuales un individuo es visto por un psiquiatra.

Q39 1. Solo estudio sobre enfermedades mentales cuando tengo que hacerlo, pero no me molestaría leer material adicional sobre las mismas.
- ❍ Totalmente de Acuerdo (1)
- ❍ De acuerdo (2)
- ❍ Algo de Acuerdo (3)
- ❍ Algo en Desacuerdo (4)
- ❍ En desacuerdo (5)
- ❍ Totalmente en Desacuerdo (6)

Q40 2. Las personas con enfermedades mentales graves nunca pueden recuperarse completamente para tener una buena calidad de vida.
- ❍ Totalmente de Acuerdo (1)
- ❍ De acuerdo (2)
- ❍ Algo de Acuerdo (3)
- ❍ Algo en Desacuerdo (4)
- ❍ En desacuerdo (5)
- ❍ Totalmente en Desacuerdo (6)

Q41 3. Trabajar en el campo de la salud mental es tan respetable como trabajar en otros campos de la salud y de la atención social.
- ❍ Totalmente de Acuerdo (1)
- ❍ De acuerdo (2)
- ❍ Algo de Acuerdo (3)
- ❍ Algo en Desacuerdo (4)
- ❍ En desacuerdo (5)
- ❍ Totalmente en Desacuerdo (6)

Q42 4. Si yo tuviera o padeciera una enfermedad mental nunca se lo haría saber a mis amigos porque tendría miedo de ser tratado de una forma diferente.
- ❍ Totalmente de Acuerdo (1)
- ❍ De acuerdo (2)
- ❍ Algo de Acuerdo (3)
- ❍ Algo en Desacuerdo (4)
- ❍ En desacuerdo (5)
- ❍ Totalmente en Desacuerdo (6)

Q43 5. Las personas con enfermedades mentales graves son más peligrosas que otras que no la tienen.

- ❍ Totalmente de Acuerdo (1)
- ❍ De acuerdo (2)
- ❍ Algo de Acuerdo (3)
- ❍ Algo en Desacuerdo (4)
- ❍ En desacuerdo (5)
- ❍ Totalmente en Desacuerdo (6)

Q44 6. El personal de la salud y de asistencia social conocen más sobre la vida de las personas tratadas por una enfermedad mental que los miembros de su familia y amigos.

- ❍ Totalmente de Acuerdo (1)
- ❍ De acuerdo (2)
- ❍ Algo de Acuerdo (3)
- ❍ Algo en Desacuerdo (4)
- ❍ En desacuerdo (5)
- ❍ Totalmente en Desacuerdo (6)

Q45 7. Si yo padeciera una enfermedad mental nunca lo admitiría a mis colegas porque tendría miedo de ser tratado de una forma diferente.

- ❍ Totalmente de Acuerdo (1)
- ❍ De acuerdo (2)
- ❍ Algo de Acuerdo (3)
- ❍ Algo en Desacuerdo (4)
- ❍ En desacuerdo (5)
- ❍ Totalmente en Desacuerdo (6)

Q46 8. Ser un profesional de la asistencia social en la salud mental no es ser un verdadero profesional de la asistencia social en la salud.

- ❍ Totalmente de Acuerdo (1)
- ❍ De acuerdo (2)
- ❍ Algo de Acuerdo (3)
- ❍ Algo en Desacuerdo (4)
- ❍ En desacuerdo (5)
- ❍ Totalmente en Desacuerdo (6)

Q47 9. Si un colega con mucha experiencia me instruye a tratar a las personas con una enfermedad mental de una manera irrespetuosa, no seguiría sus instrucciones o sus enseñanzas.

- ❍ Totalmente de Acuerdo (1)
- ❍ De acuerdo (2)
- ❍ Algo de Acuerdo (3)
- ❍ Algo en Desacuerdo (4)
- ❍ En desacuerdo (5)
- ❍ Totalmente en Desacuerdo (6)

Q48 10. Me siento cómodo lo mismo hablando con una persona con un trastorno mental que hablando con una persona con una enfermedad física.

- Totalmente de Acuerdo (1)
- De acuerdo (2)
- Algo de Acuerdo (3)
- Algo en Desacuerdo (4)
- En desacuerdo (5)
- Totalmente en Desacuerdo (6)

Q49 11. Es importante que todo profesional de la salud y asistencia social cuidando de una persona con una enfermedad mental asegure la evaluación adecuada de su salud física.

- Totalmente de Acuerdo (1)
- De acuerdo (2)
- Algo de Acuerdo (3)
- Algo en Desacuerdo (4)
- En desacuerdo (5)
- Totalmente en Desacuerdo (6)

Q50 12. La población no necesita ser protegida de las personas con una enfermedad mental grave.

- Totalmente de Acuerdo (1)
- De acuerdo (2)
- Algo de Acuerdo (3)
- Algo en Desacuerdo (4)
- En desacuerdo (5)
- Totalmente en Desacuerdo (6)

Q51 13. Si una persona con una enfermedad mental se queja de síntomas físicos (como dolor en el pecho) se lo atribuiría a su enfermedad mental.

- Totalmente de Acuerdo (1)
- De acuerdo (2)
- Algo de Acuerdo (3)
- Algo en Desacuerdo (4)
- En desacuerdo (5)
- Totalmente en Desacuerdo (6)

Q52 14. Los médicos generales no deberían esperar a completar una evaluación exhaustiva de las personas con síntomas psiquiátricos porque pueden referirlos para un psiquiatra.

- Totalmente de Acuerdo (1)
- De acuerdo (2)
- Algo de Acuerdo (3)
- Algo en Desacuerdo (4)
- En desacuerdo (5)
- Totalmente en Desacuerdo (6)

Q53 15. Yo usaría los términos “loco”, “chiflado” etc para describir con mis colegas a las personas con una enfermedad mental que veo (trato) en mi trabajo.

- Totalmente de Acuerdo (1)
- De acuerdo (2)
- Algo de Acuerdo (3)
- Algo en Desacuerdo (4)
- En desacuerdo (5)
- Totalmente en Desacuerdo (6)

Q54 16. Si un colega me dice que tiene una enfermedad mental, me gustaría continuar trabajando con él.

- Totalmente de Acuerdo (1)
- De acuerdo (2)
- Algo de Acuerdo (3)
- Algo en Desacuerdo (4)
- En desacuerdo (5)
- Totalmente en Desacuerdo (6)
