# Supplementary material for: Clinical decisions and stigmatizing attitudes towards mental health problems in primary care physicians from Latin American countries
Source: PLoS One. 2018 Nov 15;13(11):e0206440. doi: 10.1371/journal.pone.0206440 (PMC6237310; doi:10.1371/journal.pone.0206440)
Supplement: S3 Appendix — (PDF) [file pone.0206440.s003.pdf]

Primary Care Physicians Português

Q1 **Caro (a) colega convidamos você a participar neste estudo sobre as questões relativas ao tratamento dos transtornos mentais na atenção primária (básica), todas as informações fornecidas serão resguardadas na mais estrita confiança, contribuindo para o desenvolvimento de três linhas de pesquisa estão sendo desenvolvido na Universidade Federal de são Paulo (UNIFESP), Departamento de Psiquiatria, em 4 países: Brasil, Bolívia, Chile, Cuba.**

Q2. Eu sou um (a) médico (a) de atenção primária qualificado (a) para exercer no meu país, e concordo em fornecer informações no presente questionário, as quais contribuirão a melhora da atenção dos pacientes com doenças mentais na Atenção Básica da Saúde.

○ Sim (1)

○ Não (2)

Q3. Atualmente atende pacientes como parte de suas atividades profissionais?

○ Sim (1)

○ Não (2)

Q4. Gênero

○ Feminino (1)

○ Masculino (2)

Q5. Em qual cidade trabalha?

____

Q6. Nacionalidade

____

Q7. Ano de nascimento

____

Q8. Que línguas você usa, mais frequentemente, em sua vida profissional?

○ Inglês (1)

○ Português (2)

○ Espanhol (3)

Q9. Qual é a sua especialidade?

Q10. Quantos anos de treinamento profissional formal você tem, sem incluir o grau universitário? (Note que fazemos referência apenas aos anos de treino formal, não de experiência. Treino formal inclui pós-graduação, estágios de especialização ou pós-doutoramento, mas não inclui educação profissional continuada)

Q11 Como parte do seu treino profissional (acadêmico ou clínico), quais dos seguintes sistemas diagnósticos para transtornos mentais ou comportamentais você foi treinado a utilizar?

CID-10 (1)

○ CID-9 (2)

○ CID-8 (3)

○ DSM-IV (4)

○ DSM-IV-TR (5)

○ DMS-III (6)

○ Nenhum (7)

Q12. Atualmente, quais são os seus contextos de trabalho principais? Entendemos por contexto de trabalho principal o local onde trabalha cinco horas ou mais por semana. (Marque todos os que se aplicam)

- ○ Cuidados de saúde primários (1)
- ○ Outros contextos médicos gerais (incluindo centros médicos universitários, faculdade/escola de medicina) (2)
- ○ Serviços de saúde mental (incluindo serviços nas universidades, departamentos hospitalares, clínicas de ambulatórios) (3)
- ○ Programas especializados de tratamento de abuso de substâncias (4)
- ○ Consultório particular (sozinho ou em grupo) (5)
- ○ Universidade (não em serviço de tratamento médico, de saúde mental ou abuso de substâncias) (6)
- ○ Administração de saúde pública ou agência governamental (7)
- ○ Organização não-governamental (ONG) (8)
- ○ Outros (9)

Q13 Quantos anos de experiência formal tem em sua especialidade?

Q14 Por favor, indique quais dos seguintes tipos de serviços de saúde mental oferece pessoalmente a pacientes como parte das suas atividades profissionais regulares

(marque todos os que se apliquem)

- ○ Avaliação diagnóstica de transtornos mentais e comportamentais (1)
- ○ Avaliação e monitorização/gestão de substâncias psicoativas (2)
- ○ Avaliação psicológica (p.ex. avaliação neuropsicológicas, testes de personalidade, etc) (3)
- ○ Psicoterapia (4)
- ○ Intervenção psicoeducacional (5)
- ○ Outros (especifique): (6) ____
- ○ Nenhum (7)

Q15. Supervisiona diretamente os atendimentos/serviços de saúde prestados por outras pessoas? (Por supervisão direta queremos dizer que você monitora os serviços prestados através de mecanismos como supervisão face-a-face, conferência de casos, revisão de

prontuários clínicos, e que é diretamente responsável pela qualidade dos serviços clínicos que são oferecidos)

○ Sim (1)

○ Não (2)

Q16. Quantas pessoas supervisiona diretamente na prestação de serviços de saúde?

Q17. Por favor, indique quais dos seguintes tipos de serviços de saúde mental são oferecidos pelas pessoas que supervisiona? (Marque todos que se aplicam)

○ Avaliação diagnóstica de transtornos mentais e comportamentais (1)

○ Avaliação e monitorização/gestão de substâncias psicoativas (2)

○ Avaliação psicológica (p.ex. avaliação neuropsicológicas, testes de personalidade, etc.) (3)

○ Psicoterapia (4)

○ Intervenção psicoeducacional (5)

○ Nenhum (6)

Q18. Numa semana típica, em que tipo de contextos oferece ou supervisiona serviços de saúde? (Marque todos que se aplicam)

○ Serviço de Saúde Mental (1)

○ Internação (2)

○ Ambulatorial (3)

○ Domiciliar (4)

○ Hospitalização parcial/Hospital de Dia (5)

○ Clínica privada (6)

○ Consultório particular (7)

Q19. Numa semana típica, em que tipo de contextos oferece ou supervisiona serviços de saúde? (Marque todos os que se aplicam) Programa especializado para tratamento de abuso de substâncias

○ Internação (1)

○ Ambulatório (2)

○ Domiciliar (3)

○ Hospitalização parcial/Hospital de Dia (4)

○ Não se aplica (5)

Q20. Numa semana típica, em que tipo de contextos oferece ou supervisiona serviços de saúde? (Marque todos os que se aplicam)
Contextos médicos gerais

○ Hospital ou outro serviço de internação (1)

○ Ambulatório (2)

○ Domiciliar (3)

○ Hospitalización parcial/Hospital de Día (4)

○ Não se aplica (5)

Q21. Numa semana típica, em que tipo de contextos oferece ou supervisiona serviços de saúde? (Marque todos os que se aplicam)
Outros contextos:

○ Prisões, cadeias ou outros centros de detenção (1)

○ Programas comunitários ou equipes de rua (2)

○ Programas baseados nas escolas (3)

○ Centros universitários de saúde mental ou psicologia/aconselhamento (4)

○ Outros (5)

○ Não se aplica (6)

Q22. Em que tipos de comunidades estão localizados os contextos de serviço que oferece ou supervisiona? (Se oferecer serviços em mais de um ambiente, marque todos que se aplicam)

○ Grandes centros urbanos (1)

○ Subúrbio de um grande centro urbano (2)

○ Cidade de tamanho médio (3)

○ Cidade pequena (4)

Vila (5)

Ambiente rural (6)

Q23. Para os serviços de saúde que oferece ou supervisiona, qual é a percentagem de pacientes em cada grupo etário? (As percentagens têm que totalizar 100. Se alguma for zero, por favor marque)

Crianças (0-12) (1)	
Adolescentes (12-18) (2)	
Adultos (18-65) (3)	
Idosos (más de 65) (4)	

Q24. Quem é, mais frequentemente, o responsável pela atribuição do diagnóstico psiquiátrico aos pacientes que atende ou supervisiona?

Eu ou alguém sob minha supervisão direta atribui, habitualmente, o diagnóstico (1)

Outro profissional de saúde que não está sob minha supervisão atribui, habitualmente, o diagnóstico (p.ex. outro médico do serviço) (2)

Os diagnósticos são, habitualmente, atribuídos por codificadores de registros médicos (3)

Não são, habitualmente, atribuídos diagnósticos psiquiátricos (4)

Outros (5)

Q25. Por favor, indique com que regularidade utiliza os seguintes sistemas de classificação em sua prática clínica ou supervisão:

	De forma rotineira (1)	Frequentemente (2)	Às vezes (3)	Raramente (4)	Nunca (5)
CID-9 o CID-9-CM (1)	○	○	○	○	○
CID-10 (2)	○	○	○	○	○
DSM IV ou DSM-IV-TR (3)	○	○	○	○	○
Outros (4)	○	○	○	○	○

Q26 Dependendo do sistema de classificação que você usa com mais frequência na prática clínica, considera que a classificação facilita sua tarefa de diagnóstico de problemas de saúde mental em pacientes na atenção primaria?

○ Sim (1)

○ Não (2)

Q27. O sistema de classificação que você usa com mais frequência se ajusta aos sintomas que apresentam seus pacientes em sua prática clínica na atenção primária?

○ Sim (1)

○ Não (2)

Q28. Você tem responsabilidade administrativa por uma ou mais unidades que ofereçam serviços de saúde mental, mesmo que não ofereça pessoalmente cuidados clínicos diretos ou supervisão?

○ Sim (1)

○ Não (2)

Q29. **Quantas pessoas oferecem serviços de saúde mental nas unidades das quais você é responsável?**

____

Q30. Em uma semana típica, quantas horas você dedica a cada das seguintes atividades profissionais? (Se alguma for zero, por favor marque)

Fornecendo diretamente serviços de saúde mental a pacientes (p.ex. avaliações, terapias psicológicas ou comportamentais, gestão de medicação) (1)	
Fornecendo outros serviços de cuidados de saúde (não de saúde mental) (2)	
Supervisão de serviços de saúde fornecidos por outros (3)	
Ensino ou Educação (4)	
Investigação (5)	
Administração (6)	
Outros (especifique) (7)	

Q31. Por favor, relacione até três áreas relacionadas aos transtornos mentais e comportamentais da CID-10 em que tenha mais conhecimento e experiência:

○ Demência, Delirium e transtornos relacionados (1)

○ Transtornos relacionados ao uso de substâncias (2)

○ Esquizofrenias e transtornos relacionados (3)

○ Transtornos do humor (4)

○ Transtornos de ansiedade (5)

○ Transtornos relacionados ao estresse (6)

○ Transtorno obsessivo-compulsivo e transtornos relacionados (7)

○ Transtornos somatomorfes (8)

○ Transtornos alimentares (9)

○ Transtornos do sono (10)

○ Transtornos sexuais (11)

○ Transtornos de personalidade (12)

○ Deficiências intelectuais (13)

○ Transtornos do espectro do autismo (14)

○ Transtornos de déficit de atenção e de conduta (15)

○ Epidemiologia (16)

○ Saúde Pública (17)

○ Neurociência (18)

○ Outros (Especifique) (19)
____

Q32. Eu me sinto suficientemente capacitado para diagnosticar e tratar aos pacientes com transtornos mentais comuns:

○ Concordo Totalmente (1)

○ Concordo Parcialmente (2)

○ Discordo Parcialmente (3)

○ Discordo Totalmente (4)

Q33. Por favor, forneça qualquer comentário adicional sobre seus contextos de trabalho, interesses ou especialidade que considere não terem sido abordados adequadamente nesse questionário.

____

Q34. A continuação se apresentam dois casos clínicos, por favor responda as perguntas a seguir com sua conduta em relação ao caso:

Q35. **Identificação:** MDS, sexo feminino, 53 anos, chega à Unidade Básica de Saúde com queixa de "tonteira". **História Atual**: É diabética, previamente compensada. Nega náuseas, xerostomia, dor abdominal ou sintomas urinários. Diz que a tontura se iniciou na véspera, mas vem piorando progressivamente. O enfermeiro pergunta se ela "passou algum nervoso" e a paciente começa a chorar. Diz que frequentemente tem tontura, mas quando briga com o filho é pior. **Informações Adicionais:** Desde que seu filho único começou a "fazer coisas ruins por causa das más companhias", há cerca de 1 ano, está sem vontade de sair da cama. Chora várias vezes ao dia e acredita ser uma pessoa triste. Está comendo pouco, pois logo sente-se "cheia" e com náuseas, e tem muitos pesadelos. Ao examiná-la você nota que a paciente está hidratada, seus exames abdominal e

neurológico normais. A pressão arterial é de 140x90 mmHg e a glicemia de 248 mg/dl.

	Concordo Plenamente (1)	Concordo Parcialmente (2)	Discordo Parcialmente (3)	Discordo Plenamente (4)	Discordo Completamente (5)
A paciente deve ser tratada para a complicação aguda da diabetes. (1)	○	○	○	○	○
Deve ser administrado a ela um benzodiazepínico e ela pode ter alta. (2)	○	○	○	○	○
MDS pode ter um transtorno mental importante. (3)	○	○	○	○	○
O Clínico/Médico de Família e Comunidade deve tratar da diabetes e encaminhar a paciente para um psiquiatra. (4)	○	○	○	○	○
MDS apresenta um quadro de depressão que deve, a princípio, ser tratado pelo Clínico/Médico de Família. (5)	○	○	○	○	○
MDS apresenta sintomas de ansiedade que não precisam de tratamento. (6)	○	○	○	○	○
MDS tem uma depressão importante e o Clínico/Médico de Família deve iniciar o tratamento com um antidepressivo. (7)	○	○	○	○	○

Q36. **Identificação:** L.C.P., 54 anos, sexo feminino, chega para consulta marcada com queixa de "dor nos braços". **História Atual:** Diz que tem o sintoma há 6 anos, com períodos de piora, além de "crises de formigamento" em hemicorpo direito, que duram de 2 a 4 horas, pelo menos 2 vezes por semana. Refere ainda "cansaço exagerado" e períodos de empachamento gástrico. Já traz para a consulta exames realizados em diferentes serviços no último ano: hemograma, glicemia, lipemia, dosagem de eletrólitos, enzimas hepáticas e hormônios tireoidianos, endoscopia, tomografias de crânio e coluna, us doppler de artérias carótidas e vertebrais e eletro neuromiografia, todos sem alterações. **Informações Adicionais:** Há 4 anos está afastada do trabalho, desde o falecimento de seu filho. Diz que não gosta de sair de casa, não tem vontade de encontrar parentes ou amigos e só tem relações sexuais com o marido "por obrigação". Percebe que seus sintomas pioram muito quando está nervosa. Usa anti-inflamatórios não esteroidais e associações de analgésicos e relaxantes musculares quase diariamente, atualmente sem melhora significativa. Nega doenças crônicas. Na consulta, apresenta pressão arterial de 120x75mmHg, dor à palpação muscular em trapézios e romboides, sem limitação de movimentos. Exame neurológico:

força, tônus, reflexos, propriocepção e coordenação normais em membros superiores e inferiores.

	Concordo Plenamente (1)	Concordo Parcialmente (2)	Desacordo Parcialmente (3)	Desacordo Completamente (4)
A paciente deve receber uma investigação clínica mais aprofundada para uma doença orgânica. (1)	○	○	○	○
Deve ser administrado a ela um benzodiazepínico para uso contínuo. (2)	○	○	○	○
L.C.P. pode ter um transtorno mental importante. (3)	○	○	○	○
L.C.P. deve se beneficiar com o uso de antidepressivos. (4)	○	○	○	○
O Clínico/Médico de Família e Comunidade encaminhar a paciente para um psiquiatra. (5)	○	○	○	○
L.C.P. apresenta um transtorno mental que deve, a princípio, ser tratado pelo Clínico/Médico de Família. (6)	○	○	○	○
L.C.P. não apresenta doença orgânica, portanto não precisa de tratamento. (7)	○	○	○	○

Q37 **Identificação:** A.C.V., 46 anos, sexo masculino, vem para consulta com queixa de dor no peito. **História Atual:** Refere dor em aperto em hemitórax esquerdo, não relacionada com esforço, iniciada há 4 meses, com aumento progressivo na frequência desde então. Alguns destes episódios são acompanhados de dispneia, outros de parestesia em mãos. Refere ocasionalmente apresentar também tremor ou tontura. Quando questionado diz achar que tem o sono agitado. Tem se sentido irritado e brigado muito com a esposa, mas não acredita que os sintomas têm relação com seu estado emocional. **Informações Adicionais:** Conta que tem um filho de 24 anos que vai se casar em 1 mês e que sua filha de 19 anos quer entrar na faculdade, mas não sabe se vai conseguir pagar. Hipertenso, em uso de Hidroclorotiazida 25mg pela manhã e Enalapril 5mg a cada 12 horas, nega história familiar de doenças cardiovasculares. Comerciante, trabalha cerca de 12 horas por dia, nega tabagismo. Ao exame: pressão arterial 110x70, peso 75kg, altura 1,68m (IMC 25,5), ausculta cardíaca e pulmonar sem alterações. Traz resultados de exames complementares realizados no mês anterior: glicemia 99 mg/dl, colesterol total 223, HDL

65, triglicerídeos 145, creatinina 0,9, potássio 3,8, urina sem proteinúria e eletrocardiograma com ritmo sinusal, sem anormalidades.

	Concordo Plenamente (1)	Concordo Parcialmente (2)	Disacordo Parcialmente (3)	Disacordo Completamente (4)
O paciente precisa apenas de investigação para síndrome coronariana. (1)	○	○	○	○
O paciente tem indicação de uso de um antidepressivo. (2)	○	○	○	○
A.C.V. tem indicação de uso de um benzodiazepínico. (3)	○	○	○	○
O Clínico/Médico de Família e Comunidade deve encaminhar o paciente para um psiquiatra. (4)	○	○	○	○
A.C.V. apresenta um quadro de ansiedade que deve, a princípio, ser tratado pelo Clínico/Médico de Família. (5)	○	○	○	○
A.C.V. não precisa de nenhum tipo de tratamento além do atual para hipertensão. (6)	○	○	○	○

Q39 1. Só estudo sobre saúde mental quando necessito, mas não me incomodaria em ler material adicional sobre o assunto.

- Condordo Plenamente (1)
- Concordo Parcialmente (2)
- Concordo (3)
- Discordo Parcialmente (4)
- Discordo (5)
- Discordo Plenamente (6)

Q40 2. Pessoas com quadro mental grave nunca podem se recuperar o suficiente para ter uma boa qualidade de vida.

- Concordo Plenamente (1)
- Concordo Parcialmente (2)
- Concordo (3)
- Discordo Parcialmente (4)
- Discordo (5)
- Discordo Plenamente (6)

Q41 3.Trabalhar no campo da saúde mental é tão respeitável quanto em outros campos da saúde e da atenção social.

- Concordo Plenamente (1)
- Concordo Parcialmente (2)
- Concordo (3)
- Discordo Parcialmente (4)
- Discordo (5)
- Discordo Plenamente (6)

Q42 4. Se eu tivesse uma doença mental nunca admitiria isso para meus amigos porque eu teria medo de ser tratado de modo diferente.

- Concordo Plenamente (1)
- Concordo Parcialmente (2)
- Concordo (3)
- Discordo Parcialmente (4)
- Discordo (5)
- Discordo Plenamente (6)

Q43 5. Pessoas com transtorno mental grave são geralmente mais perigosos que os demais.

- Concordo Plenamente (1)
- Concordo Parcialmente (2)
- Concordo (3)
- Discordo Parcialmente (4)
- Discordo (5)
- Discordo Plenamente (6)

Q44 6.Profissionais da saúde e do serviço social conhecem mais sobre a vida das pessoas tratadas por doença mental do que os membros da família ou amigos.

- Concordo Plenamente (1)
- Concordo Parcialmente (2)
- Concordo (3)
- Discordo Parcialmente (4)
- Discordo (5)
- Discordo Plenamente (6)

Q45 7. Se eu tivesse uma doença mental eu nunca admitiria isso aos meus colegas de trabalho por medo de ser tratado de modo diferente.

- Concordo Plenamente (1)
- Concordo Parcialmente (2)
- Concordo (3)
- Discordo Parcialmente (4)
- Discordo (5)
- Discordo Plenamente (6)

Q46 8.Ser um profissional da saúde ou do serviço social na área da saúde mental não é ser um verdadeiro profissional da saúde ou do serviço social.

- Concordo Plenamente (1)
- Concordo Parcialmente (2)
- Concordo (3)
- Discordo Parcialmente (4)
- Discordo (5)
- Discordo Plenamente (6)

Q47 9. Se um colega com muita experiência me instrui a tratar as pessoas com uma doença mental de uma maneira desrespeitosa, não seguiria suas instruções.

- Concordo Plenamente (1)
- Concordo Parcialmente (2)
- Concordo (3)
- Discordo Parcialmente (4)
- Discordo (5)
- Discordo Plenamente (6)

Q48 10. Eu me sinto tão confortável em conversar com uma pessoa com doença mental quanto conversar com uma pessoa com uma doença física.

- Concordo Plenamente (1)
- Concordo Parcialmente (2)
- Concordo (3)
- Discordo Parcialmente (4)
- Discordo (5)
- Discordo Plenamente (6)

Q49 11. É importante que qualquer profissional da saúde ou de assistência social quando der suporte a uma pessoa com uma doença mental também garanta que sua saúde física seja avaliada.

- Concordo Plenamente (1)
- Concordo Parcialmente (2)
- Concordo (3)
- Discordo Parcialmente (4)
- Discordo (5)
- Discordo Plenamente (6)

Q50 12. A população não precisa ser protegida das pessoas com uma doença mental grave.

- Concordo Plenamente (1)
- Concordo Parcialmente (2)
- Concordo (3)
- Discordo Parcialmente (4)
- Discordo (5)
- Discordo Plenamente (6)

Q51 13. Se uma pessoa com uma doença mental se queixar de um sintoma físico (como uma dor no peito) eu atribuiria isso a sua doença mental.

- Concordo Plenamente (1)
- Concordo Parcialmente (2)
- Concordo (3)
- Discordo Parcialmente (4)
- Discordo (5)
- Discordo Plenamente (6)

Q52 14. Clínicos gerais não deveriam esperar realizar uma avaliação mais completa de pessoas com sintomas psiquiátricos já que eles poderiam ser encaminhados a um psiquiatra.

- Concordo Plenamente (1)
- Concordo Parcialmente (2)
- Concordo (3)
- Discordo Parcialmente (4)
- Discordo (5)
- Discordo Plenamente (6)

Q53 15. Eu usaria os termos “louco”, “doido”, “maluco” etc. para descrever para colegas de profissão as pessoas com doença mental que atendo no meu trabalho.

- ◯ Concordo Plenamente (1)
- ◯ Concordo Parcialmente (2)
- ◯ Concordo (3)
- ◯ Discordo Parcialmente (4)
- ◯ Discordo (5)
- ◯ Discordo Plenamente (6)

Q54 16. Se um colega me conta que ele tem uma doença mental, eu seguiria trabalhando com ele.

- ◯ Concordo Plenamente (1)
- ◯ Concordo Parcialmente (2)
- ◯ Concordo (3)
- ◯ Discordo Parcialmente (4)
- ◯ Discordo (5)
- ◯ Discordo Plenamente (6)
